# Depression, anxiety, and burnout among psychiatrists during the COVID-19 pandemic: a cross-sectional study in Beijing, China

**DOI:** 10.1186/s12888-023-04969-5

**Published:** 2023-07-10

**Authors:** Ping Dong, Xiao Lin, Fei Wu, Sijia Lou, Na Li, Sifan Hu, Le Shi, Jia He, Yundong Ma, Yanping Bao, Lin Lu, Wei Sun, Hongqiang Sun

**Affiliations:** 1 NHC Key Laboratory of Mental Health (Peking University), Peking University Sixth Hospital, Peking University Institute of Mental Health, National Clinical Research Center for Mental Disorders (Peking University Sixth Hospital, Peking University), Beijing, 100191 China; 2 National Institute on Drug Dependence and Beijing Key Laboratory on Drug Dependence Research, Peking University, Beijing, 100191 China; 3 Peking-Tsinghua Center for Life Sciences and PKU-IDG, McGovern Institute for Brain Research, Beijing, 100191 China

**Keywords:** Psychiatrist, Mental health, Depressive symptoms, Anxiety symptoms, Burnout, COVID-19

## Abstract

**Background:**

With the rise of reported mental disorders and behavioral issues after the outbreak of the coronavirus disease 2019 (COVID-19) pandemic, psychiatrists and mental health care are urgently needed more than ever before. The psychiatric career carries a high emotional burden and stressful demands, which bring issues on psychiatrists’ mental health and well-being into question. To investigate the prevalence and risk factors of depression, anxiety, and work burnout among psychiatrists in Beijing during the COVID-19 pandemic.

**Methods:**

This cross-sectional survey was conducted from January 6 to January 30, 2022, two years after COVID-19 was declared a global pandemic. Recruitment was performed using a convenience sample approach by sending online questionnaires to psychiatrists in Beijing. The symptoms of depression, anxiety, and burnout were evaluated using the Patient Health Questionnaire-9 (PHQ-9), Generalized Anxiety Disorder-7 (GAD-7), and Maslach Burnout Inventory-General Survey (MBI-GS). The perceived stress and social support were measured by the Chinese Perceived Stress Scale (CPSS) and Social Support Rating Scale (SSRS), respectively.

**Results:**

The data of 564 psychiatrists (median [interquartile range] age, 37 [30–43] years old) of all 1532 in Beijing were included in the statistical analysis. The prevalence of symptoms of depression, anxiety and burnout were 33.2% (95% CI, 29.3-37.1%, PHQ-9 ≥ 5), 25.4% (95% CI, 21.8-29.0%, GAD-7 ≥ 5) and 40.6% (95% CI, 36.5-44.7%, MBI-GS ≥ 3 in each of the three subdimensions), respectively. The psychiatrist with a higher score on perceived stress was more likely to suffer from depressive symptoms (adjusted odds ratios [ORs]: 4.431 [95%CI, 2.907–6.752]); the anxiety symptoms (adjusted ORs: 8.280 [95%CI, 5.255–13.049]), and the burnout conditions (adjusted ORs: 9.102 [95%CI, 5.795–14.298]). Receiving high social support was an independent protective factor against symptoms of depression (adjusted ORs: 0.176 [95%CI, [0.080–0.386]), anxiety (adjusted ORs: 0.265 [95%CI, 0.111–0.630]) and burnout (adjusted ORs: 0.319 [95%CI, 0.148–0.686]).

**Conclusions:**

Our data suggest a considerable proportion of psychiatrists also suffer from depression, anxiety, and burnout. Perceived stress and social support influence depression, anxiety, and burnout. For public health, we must work together to reduce the pressure and increase social support to mitigate mental health risks in psychiatrists.

**Supplementary Information:**

The online version contains supplementary material available at 10.1186/s12888-023-04969-5.

## Introduction

Mental health issues have been one of the top ten leading causes of global disease burden, with no evidence of a reduction in the burden since 1990 [1]. There was a high prevalence of symptoms of depression, anxiety, insomnia, and acute stress during the COVID-19 pandemic [2]. Psychiatrists are needed more than ever to optimize the population’s mental health by providing interventions for outbreak-related distress responses [3]. Increased work hours and workloads are common to meet the increasing mental health service demand during the COVID-19 pandemic. During this period, all healthcare workers, including psychiatrists, were affected by various challenges. Besides the routine work, they must educate health care and community leaders about effective ways to support the population’s mental health during the COVID-19 outbreak. They have to respond to the challenges of the epidemic prevention and control requirements and the pressure of increased numbers of patients. Some mental hospitals must implement weekly or even monthly shift duty, meaning the psychiatrists must stay there for a week or even a month. In addition, due to a shortage of medical staff, they have to be rotated to the epidemic area to support large-scale nucleic acid testing work. However, there are not enough psychiatrists to meet this demand [4], and this shortage is more severe in China [5, 6].

In recent years, China has gradually strengthened the construction of the mental health prevention and control system, forming progressively a national, provincial, prefecture-level city, county (district), township (street), village (community) mental health prevention and control service system that is mainly composed of specialized psychiatric hospitals, supplemented by comprehensive hospitals, supported by primary medical and health institutions and mental disease rehabilitation institutions, and supplemented by disease prevention and control centers [7]. The National Health Commission of the People’s Republic of China has set standards for different levels of psychiatric hospitals (http://www.nhc.gov.cn), and the standard considered several aspects, such as the number of beds and medical workers. Psychiatrists play the most crucial role in guiding the diagnosis and treatment of mental illnesses, but cultivating a psychiatrist is a long process. In China, to train as a psychiatrist, you will start training as a medical doctor (4–6 years), then receive standardized training for resident physicians, according to different majors, including psychiatry. After a year of “24-hour resident physician” training, you can become a psychiatrist, but completing the training requires at least three years. There is a national psychiatrist certification system, and every psychiatrist has to pass this qualification to practice psychiatry. This process usually takes at least 6 years from receiving medical education. Numerous studies have addressed the escalating shortage of psychiatrists, and several programs have tried recruiting future psychiatrists [8, 9]. To increase the supply of the psychiatric workforce, 31 medical colleges across China have established psychiatry programs for medical students with vigorous promotion by the National Health Commission and the Ministry of Education. These are specialized programs with a curriculum focusing on psychiatry-related teaching, to fast-track graduates to psychiatry residency directly. Compared to increasing the number of future psychiatrists, the more urgent problem is investigating the mental health and work burnout status of practicing psychiatrists to improve their health status and better serve patients.

Psychiatrists have basic similarities with other professional doctors but may also have some unique characteristics. For example, a study proposed that good psychiatrists were defined as “a good communicator and listener with a professional manner, who respects confidentiality and has good doctor-patient relationships“ [10]. In addition, long-term contact with people with mental health conditions has great mental pressure and practicing risk [11]. The economic income of psychiatrists is relatively low [12], the social appreciation is unrecognized, and there is a certain sense of practicing shame, etc., which may cause high emotional burdens [13]. Besides, the high probability of experiencing patient suicide may be a common source of distress for psychiatrists [14]. A previous study reported that 5% of psychiatry trainees in the United States experienced a patient suicide within one year and estimated that 20% would experience it at least once over their traineeship [15]. Compared with doctors from other specialties, psychiatrists are more likely to suffer from various mental health problems [16]. Longitudinal studies in the UK also suggest that psychiatrists suffer from exceptionally high levels of stress and the highest levels of depression [17].

The symptoms of depression and anxiety are the most frequently reported mental health issues in health careers, including psychiatrists. The definition of depression is based on symptoms forming a syndrome, including depressed mood, anhedonia, feelings of worthlessness or guilt, suicidal ideation, plan, or attempt, fatigue or loss of energy, insomnia or increased sleep, loss or increase in appetite or weight and so on [18]. Anxiety often has a series of symptoms, such as “feeling keyed up or tense”, “being unusually restless”, “having trouble concentrating because of worry”, “having a fear that something awful may happen”, and “feeling of that one might lose control of oneself” [19].Anxiety is common in the context of depression, and almost two-thirds of individuals with major depressive disorder have clinical anxiety [20].Another severe condition of a psychiatrist is work burnout [21], which threatens the psychiatrist’s health and leads to performance inefficiency and despair. Burnout is a psychological syndrome in response to chronic occupational stressors, consisting of three key dimensions: overwhelming exhaustion, depersonalization, and low personal accomplishment [22]. Burnout is closely related to depression and anxiety in previous study [23], and these symptoms often co-occur among medical staff [24, 25]. However, burnout and clinical symptoms of depression and anxiety were empirically distinct, highlighting the importance of screening for burnout and clinical symptoms to allow fast access to adequate support and treatment in health professionals [26]. Before the COVID-19 pandemic, a study investigated the prevalence of anxiety and depression among hospital psychiatrists and reported that the prevalence of depression and anxiety was 22.5% and 28.1% in 285 psychiatrists [27]. A systematic review and meta-analysis reported a pooled prevalence of 23.2% for anxiety and 22.8% for depression in healthcare workers during the outbreak of COVID-19 [28]. A later meta-analysis only included data from China revealed a higher prevalence of anxiety (31.5%) and depression (23.7%) in healthcare workers [29]. While the psychiatrists’ career carries a high emotional burden and stressful demands, there is a lack of studies estimating the prevalence and evaluating the risk factors related to the mental health of psychiatrists in China. A recent study reported that the anxiety level in psychiatrists is lower than in physicians [27]. A recent study in healthcare reported no significant increase in anxiety symptoms after the COVID-19 pandemic [30]. Previous studies have shown that the overall burnout among physicians during the outbreak of the COVID-19 pandemic ranged from 14.7–90.4% [31], and the introduction of COVID-19 has heightened existing challenges that physicians face, such as increased workload, which is directly correlated with increased burnout [32].

This study aims to investigate the prevalence and risk factors of depression, anxiety, and work burnout among psychiatrists in Beijing during the COVID-19 pandemic.The present study surveyed psychiatrists’ mental health for several reasons. First, the psychiatrist-patient relationship is unique among medical specialties, as psychiatrists become “tools” in their profession. Second, most psychiatrists experience stressful adversities such as attacks by violent patients or hostile relatives of patients and experiencing patient suicide. These conditions increased the emotional burdens of psychiatrists. Furthermore, the shortage of psychiatrists may cause them to neglect their personal care by saturating their time with multiple consultations. The need for psychiatric care in China rapidly increases due to economic and social development and population aging, especially during the COVID-19 epidemic. Besides recruiting more future psychiatrists, the retention issues of currently practicing psychiatrists are more urgent in China. Considering the lingering effect of COVID-19 on people’s mental health and the importance of psychiatrists in this process, the survey was conducted in January 2022, two years after COVID-19 was declared a global pandemic. In this particular month, 31 province-level regions in China have reported 3634 new confirmed COVID-19 cases, and Beijing has reported 135 new confirmed COVID-19 cases (http://www.nhc.gov.cn/; http://wjw.beijing.gov.cn/).

## Methods

### Study design and participants

A cross-sectional survey was conducted in Beijing, China, from January 6 to January 30, 2022. The study was approved by the ethics committee of Peking University Sixth Hospital (Institute of Mental Health). Written informed consent was received before the respondents began the questionnaire. This study follows the American Association for Public Opinion Research (AAPOR) reporting guideline. A self-designed survey was initiated by The Beijing Medical Doctor Association, a voluntary, non-profit medical professional association in China. This online survey is published and collected through “Questionnaire Star” (https://www.wjx.cn/). Recruitment was performed using a convenience sample approach. Based on the feasibility of the research, the simple sampling method we used was similar to stratified sampling, but it was not strictly a typical stratified sampling. The survey was sent to the psychiatric specialist hospitals and general hospitals with a psychiatric departments at all levels in Beijing through the psychiatry specialist branch of the Beijing Medical Doctor Association. The psychiatric system consists of several levels of medical institutions: Primary, Secondary, and Tertiary (the National Health Commission set the standard). The contact person of the hospital forwarded the questionnaire to their respective hospital’s doctor work WeChat group, explaining the purpose of the survey and notifying the doctors to fill it out voluntarily. Each network device can only fill in the questionnaire once. This was an anonymous survey, and the confidentiality of the data was ensured. This survey was designed to investigate the mental health of practicing psychiatrists in Beijing, China. The inclusion criteria: [1] voluntarily participate; [2] aged 18–65 years old; [3] working in medical institutions in Beijing now. The exclusion criteria: a history of mental illness that met the criteria on DSM-5, such as schizophrenia, bipolar disorder, depressive disorder, anxiety disorder, obsessive-compulsive disorder, insomnia disorder, alcohol dependence, or drug dependence, existed before engaging in psychiatric work. The target sample size was determined by assuming that the prevalence (p) of depression symptoms would be 28.8% [33]. We selected the depressive symptoms to estimate the sample size for two reasons. First, regarding the disease classification level of mental disorders, the disease level of depression is higher than that of anxiety and burnout, and depression often has more severe consequences. Second, a larger sample size with a lower detection rate is needed, so we calculated the sample size according to the symptoms with the lowest detection rate [24]. We used G*power to calculate the required sample size (n = 422). Based on a 75% response rate assumption, this would require surveying 563 individuals.

### Measurements and instruments

The questionnaire consists of three sections with 103 items. The first part gathered demographic information of the participants, including gender, age, marital status, level of education, and annual income. The second part included questions about the professional lives: professional title; category, and level of the medical institution; the major sub-discipline in psychiatry; working address; working years in psychiatry; working days per week; daily working hours; the number of beds in charge; providing psychological counseling or not; and the forms of assaults that have been suffered. The information about these 2 parts is listed in eTable 1 in the Supplement. The survey also included questions on mental health status, including 5 standardized scales, including the Chinese versions of Patient Health Questionnaire-9 (PHQ-9) [34], Generalized Anxiety Disorder-7 (GAD-7) [35], Maslach Burnout Inventory-General Survey (MBI-GS) [36], Chinese Perceived Stress Scale (CPSS) [37],and Social Support Rating Scale (SSRS) [38], which measured symptoms of depression, anxiety, burnout, perceived stress, and social support respectively. The total scores of these scales were interpreted as follows: PHQ-9, normal (0–4), mild [5–9], moderate [10–14], and severe [15–27] depression; GAD-7, normal (0–4), mild [5–9], moderate [10–14], and severe [15–21] anxiety; MBI-GS (including three dimensions, Emotional Exhaustion, Cynicism, Reduced Personal Accomplishment, each range 0–6, with a higher score indicating more burnout), normal (< 3 on each of the three dimensions), mild (≥ 3 in one of the three dimensions), moderate (≥ 3 in two of the three dimensions), and severe (≥ 3 in three dimensions). CPSS, scored 0–56, with a higher score indicating perceived higher stress. SSRS [39], scored 13–66, with a higher score meaning a higher level of social support the participant received. In the present study, cutoff scores of 5 for the PHQ-9, 5 for the GAD-7, and 3 in each dimension for burnout were adopted to detect depression, anxiety, and burnout symptoms. In addition, we divided perceived stress into low and high levels with 25 as the boundary points and social support into low, moderate, and high groups with 31 and 49 as the boundary points to analyze the impact on anxiety, depression, and burnout [40]. The Cronbach’s Alpha of the measurements is: PHQ-9: 0.894; GAD-7: 0.918; MBI-GS: 0.923; CPSS: 0.863; SSRS: 0.717.

## Statistical analysis

In the initial step of our analysis, we used descriptive statistics to present the demographic characteristics of the included psychiatrists. The prevalence of depression, anxiety, and burnout status was calculated using the aforementioned cutoff scores and reported as the percentages of cases in different populations. The exact binomial methods produced 95%CIs. The Chi-square test was used to compare the differences in prevalence rates among different categories. Then, we used multivariable logistic regression models to explore which factors were associated with depression, anxiety, and burnout; regression coefficient (B), odds ratios (ORs) and 95%CIs are presented. The intent of these models was explanatory and illustrative rather than predictive, and detailed information on variables in the logistic regression is listed in eTable 2 in the Supplement. All the variables statistically significant in the Chi-square test were entered into the multivariable model. *P* values were 2-sided and considered statistically significant at less than 0.05. Analyses were conducted using SPSS statistical software version 22.0 (IBM Corp). Preliminary analyses were conducted in February 2022, and final examinations were conducted in June 2022.

## Results

### Demographic characteristics

In total, 695 individuals working in different medical institutions from 14 districts in Beijing signed the informed consent, and 14 did not agree to participate in this study. Of the 681 participants, 71 were not psychiatrists, 37 had a history of mental illness before, and 9 of them did not pass the data quality examination (1 person filled in the working years exceeding his age, 7 persons filled in the working days per week exceeding 7 days, and 1 person filled in the operating hours per day exceeding 24 h ). Finally, the data of 564 psychiatrists were included in the study (see Table 1 for detailed information). Of the sample included in the data analysis, 383 (67.9%) participants were female, and the median (interquartile range) age was 37 [30–43] years; 412 (73.0%) were married, and 350 (62.1%) have children. Among the participating psychiatrists, all of them had a Junior college degree or higher, and more than half of them have a moderate annual income (342, 60.6% make about 14,360–43,080 $ per year). Besides, 134 (23.8%) of the psychiatrists have a senior professional title; 444 (78.7%) work at a psychiatric hospital; 348 (61.7%) work at a principal tertiary care hospital; most of them (371, 65.8%) major in general psychiatry; about half of them (296, 52.5%) working in the field of psychiatry for above 10 years; 324 (57.4%) work more than 8 h per day; 238 (42.2%) were in charge of at least 15 hospitalized patients, and 246 (43.6%) of them treated at least 15 outpatients in half a day. Notably, 513 (91%) of the psychiatrists have been assaulted at least once, including verbal threats, attacked with or without instruments, or other forms of assault by the patients or their relatives.

**Table 1:** Demographic and occupational characteristics of the total Sample

Items	Participants, NO. (%)
Overall	564(100.0)
Gender	
Male	181(32.1)
Female	383(67.9)
Age, y	
23–35	256(45.4)
36–45	190(33.7)
46–65	118(20.9)
Marital status	
Married	412(73.0)
Unmarried or Divorced	152(27.0)
Having children or not	
Yes	350(62.1)
Educational level	
Bachelor degree or below	322(57.1)
Master degree	175(31.0)
Doctoral degree	67(11.9)
Annual income, $	
≤ 14,360	157(27.8)
> 14,360 ≤ 43,080	342(60.6)
> 43,080	65(11.5)
Professional title	
Junior professional title	228(40.4)
Medium professional title	202(35.8)
Senior professional title	134(23.8)
Category of medical institution	
Psychiatric hospital	444(78.7)
Other medical institutions	120(21.3)
Level of medical institution	
Primary medical institutions	46(8.2)
Secondary hospital	170(30.1)
Tertiary hospital	348(61.7)
Major in psychiatry	
General psychiatry	371(65.8)
Geriatric psychiatry	59(10.5)
Pediatric psychiatry	19(3.4)
Addictive psychiatry	15(2.7)
Mental rehabilitation	25(4.4)
Psychosomatic medicine	22(3.9)
Sleep medicine	12(2.1)
Emergency psychiatry	13(2.3)
Others	28(5.0)
Working years in psychiatry, y	
≤ 10	296(52.5)
> 10	268(47.5)
Working days per week, d	
≤ 5	426(75.5)
> 5	138(24.5)
Daily working hours, h	
≤ 8	324(57.4)
> 8	240(42.6)
Number of beds in charge	
0	126(22.3)
1–15	200(35.5)
> 15	238(42.2)
Number of outpatients treated in half a day	
0	223(39.5)
1–15	95(16.8)
> 15	246(43.6)
Providing psychological counseling or not	
No	409(72.5)
Yes	155(27.5)
Forms of attacks that have been suffered	
Never been attacked	51(9.0)
Suffered only one form of attack	255(45.2)
Suffered two forms of attack	214(37.9)
Suffered three or more forms of attack	44(7.8)

### Prevalence of depressive and anxiety symptoms and burnout rate in psychiatrists

The prevalence of symptoms for the three mental health conditions was 33.2% (95%CI, 29.3-37.1%) for depression (187 participants total, including 137 participants [24.3%] with mild depression and 50 participants [8.9%] with moderate-to-severe depression), 25.4% (95%CI, 21.8-29.0%) for anxiety (143 participants total, including 116 participants [20.6%] with mild anxiety and 27 [4.8%] with moderate-to-severe anxiety), and 40.6% (95%CI, 36.5-44.7%) for burnout (229 participants total, including 160 participants [28.4%] with mild burnout and 69 participants [12.2%] with moderate-to-severe burnout). We also used *χ*^*2*^ tests to compare the prevalence of depression, anxiety, and burnout in different populations (for details, see Table 2). The prevalence of symptoms of the 3 mental health was high among psychiatrists with low income (depression, 44.6%; anxiety, 32.5%; burnout, 51.0%), high perceived stress (depression, 60.2%; anxiety, 57.1%; burnout, 78.3%), and low social support (depression, 60.0%; anxiety, 46.3%; burnout, 67.5%). The prevalence of symptoms of depression was high among psychiatrists in males (39.8%), with Bachelor’s degree or below (37.9%), working in secondary hospitals (41.8%), worked in the field of psychiatry for more than 10 years (37.3%), treating 0 patients in half a day (38.1%), and being assaulted by three or more forms (45.5%). The prevalence of symptoms of anxiety was high among psychiatrists in males (31.5%), having children (28.3%), working in a psychiatric hospital (27.3%), working in secondary hospitals (30.6%), working more than 8 h per day (29.6%), and being assaulted by three or more forms (40.9%). The prevalence of burnout was high among psychiatrists aged 18–35 years (47.7%), having no children (48.6%), with a primary title (52.2%), worked less than 10 years (46.3%), treating 0 patients in half a day (50.2%).

**Table 2:** Prevalence of Symptoms of Depression, Anxiety, and Burnout among Psychiatrists in Beijing, China

		Depression								Anxiety											Burnout									
	Participants, NO. (%)								Participants, NO. (%)											Participants, NO. (%)										
Variables		Mild	Moderate to severe	Total, No. (%)[95%CI]			*P* value	Mild			Moderate to severe		Total, No. (%)[95%CI]				*P* value		Mild			Moderate to severe		Total, No. (%)[95%CI]				*P* value		
Overall		137(24.3)	50(8.9)	187(33.2)[29.3–37.1]				116(20.6)			27(4.8)		143(25.4)[21.8–29.0]						160(28.4)			69(12.2)		229(40.6)[36.5–44.7]						
Gender																														
Male		54(29.8)	18(9.9)	72(39.8)[32.6–47.0]			0.022	45(24.9)			12(6.6)		57(31.5)[24.7–38.3]				0.021		53(29.3)			25(13.8)		78(43.1)[35.8–50.4]				0.408		
Female		83(21.7)	32(8.4)	115(30.0)[25.4–34.6]				71(18.5)			15(3.9)		86(22.5)[18.3–26.7]						107(27.9)			44(11.5)		151(39.4)[34.5–44.3]						
Age, y																														
18–35		61(23.8)	19(7.4)	80(31.3)[25.5–37.0]			0.149	47(18.4)			11(4.3)		58(22.7)[17.5–27.8]				0.403		82(32.0)			40(15.6)		122(47.7)[41.5–53.8]^a^				0.008		
36–45		40(21.1)	19(10.0)	59(31.1)[24.4–37.7]				43(22.6)			9(4.7)		52(27.4)[21.0-33.8]						49(25.8)			18(9.5)		67(35.3)[28.4–42.1]^b^						
46–65		36(30.5)	12(10.2)	48(40.7)[31.7–49.7]				26(22.0)			7(5.9)		33(28.0)[19.7–36.2]						29(24.6)			11(9.3)		40(33.9)[25.2–42.6]^b^						
Marital status																														
Married		98(23.8)	36(8.7)	134(32.5)[28.0-37.1]			0.600	87(21.1)			19(4.6)		106(25.7)[21.5–30.0]				0.737		118(28.6)			44(10.7)		162(39.3)[34.6–44.1]250				0.307		
Unmarried or Divorced		39(25.7)	14(9.2)	53(34.9)[27.2–42.5]				29(19.1)			8(5.3)		37(24.3)[17.4–31.2]						42(27.6)			25(16.4)		67(44.1)[36.1–52.1]						
Having children or not																														
No		55(25.7)	18(8.4)	73(34.1)[27.7–40.5]			0.706	35(16.4)			9(4.2)		44(20.6)[15.1–26.0]				0.041		71(33.2)			33(15.4)		104(48.6)[41.8–55.3]				0.003		
Yes		82(23.4)	32(9.1)	114(32.6)[27.6–37.5]				81(23.1)			18(5.1)		99(28.3)[23.5–33.0]						89(25.4)			36(10.3)		125(35.7)[30.7–40.8]						
Educational level																														
Bachelor degree or below		92(28.6)	30(9.3)	122(37.9)[32.6–43.2]^a^			0.022	72(22.4)			16(5.0)		88(27.3)[22.4–32.2]				0.460		92(28.6)			41(12.7)		133(41.3)[35.9–46.7]				0.401		
Master degree		33(18.9)	15(8.6)	48(27.4)[20.8–34.1]^a^				33(18.9)			7(4.0)		40(22.9)[16.6–29.1]						46(26.3)			19(10.9)		65(37.1)[29.9–44.4]						
Doctoral degree		12(17.9)	5(7.5)	17(25.4)[14.7–36.1]^a^				11(16.4)			4(6.0)		15(22.4)[12.1–32.6]						22(32.8)			9(13.4)		31(46.3)[34.0-58.5]						
Annual income, $																														
≤ 14,360		53(33.8)	17(10.8)	70(44.6)[36.7–52.4]^a^			0.001	42(26.8)			9(5.7)		51(32.5)[25.1–39.9]^a^				0.032		51(32.5)			29(18.5)		80(51.0)[43.0-58.9]^a^				0.006		
> 14,360 ≤ 43,080		66(19.3)	27(7.9)	93(27.2)[22.5–31.9]^b^				60(17.5)			14(4.1)		74(21.6)[17.3–26.0]^b^						93(27.2)			35(10.2)		128(37.4)[32.3–42.6]^b^						
> 43,080		18(27.7)	6(9.2)	24(36.9)[24.9–49.0]^ab^				14(21.5)			4(6.2)		18(27.7)[16.5–38.9]^ab^						16(24.6)			5(7.7)		21(32.3)[20.6–44.0]^b^						
Professional title																														
Primary title		61(26.8)	20(8.8)	81(35.5)[29.3–41.8]			0.173	44(19.3)			13(5.7)		57(25.0)[19.3–30.7]				0.985		81(35.5)			38(16.7)		119(52.2)[45.7–58.7]^a^				< 0.001		
Intermediate title		38(18.8)	19(9.4)	57(28.2)[22.0-34.5]				43(21.3)			9(4.5)		52(25.7)[19.7–31.8]						42(20.8)			21(10.4)		63(31.2)[24.7–37.6]^b^						
Senior title		38(28.4)	11(8.2)	49(36.6)[28.3–44.8]				29(21.6)			5(3.7)		34(25.4)[17.9–32.8]						37(27.6)			10(7.5)		47(35.1)[26.9–43.3]^b^						
Category of medical institution																														
Psychiatric hospital		113(25.5)	42(9.5)	155(34.9)[30.5–39.4]			0.089	100(22.5)			21(4.7)		121(27.3)[23.1–31.5]				0.046		131(29.5)			56(12.6)		187(42.1)[37.5–46.7]				0.159		
Other medical institutions		24(20.0)	8(6.7)	32(26.7)[18.6–34.7]				16(13.3)			6(5.0)		22(18.3)[11.3–25.4]						29(24.2)			13(10.8)		42(35.0)[26.3–43.7]						
Level of medical institution																														
Primary medical institutions		7(15.2)	3(6.5)	10(21.7)[9.4–34.1]^a^			0.009	4(8.7)			2(4.3)		6(13.0)[2.9–23.2]^a^				0.043		11(23.9)			3(6.5)		14(30.4)[16.6–44.2]				0.341		
Secondary hospital		56(32.9)	15(8.8)	71(41.8)[34.3–49.3]^b^				47(26.7)			5(2.9)		52(30.6)[23.6–37.6]^a^						50(29.4)			21(12.4)		71(41.8)[34.3–49.3]						
Tertiary hospital		74(21.3)	32(9.2)	106(30.5)[25.6–35.3]^a^				65(18.7)			20(5.7)		85(24.4)[19.9–29.0]^a^						99(28.4)			45(12.9)		144(41.4)[36.2–46.6]						
Working years in psychiatry, y																														
≤ 10		67(22.6)	20(6.8)	87(29.4)[24.2–34.6]			0.046	51(17.2)			15(5.1)		66(22.3)[17.5–27.1]				0.079		96(34.2)			41(13.9)		137(46.3)[40.6–52.0]				0.004		
> 10		70(26.1)	30(11.2)	100(37.3)[31.5–43.1]				65 (24.3)			12(4.5)		77(28.7)[23.3–34.2]						64(23.9)			28(10.4)		92(34.3)[28.6–40.0]						
Working days per week, d																														
≤ 5		106(24.9)	33(7.7)	139(32.6)[28.2–37.1]			0.640	86(20.2)			19(4.5)		105(24.6)[20.5–28.8]				0.498		129(30.3)			48(11.3)		177(41.5)[36.9–46.2]				0.421		
> 5		31(22.5)	17(12.3)	48(34.8)[26.7–42.8]				30(21.7)			8(5.8)		38(27.5)[20.0-35.1]						31(22.5)			21(15.2)		52(37.7)[29.5–45.9]						
Daily working hours, h																														
≤ 8		77(23.8)	23(7.1)	100(30.9)[25.8–35.9]			0.179	63(19.4)			9(2.8)		72(22.2)[17.7–26.8]				0.047		95(29.3)			26(8.0)		121(37.3)[32.1–42.6]				0.067		
> 8		60(25.0)	27(11.3)	87(36.3)[30.1–42.4]				53(22.1)			18(7.5)		71(29.6)[23.8–35.4]						65(27.1)			43(17.9)		108(45.0)[38.7–51.3]						
Number of beds in charge																														
0		25(19.8)	11(8.7)	36(28.6)[20.6–36.6]			0.387	21(16.7)			8(6.3)		29(23.0)[15.6–30.5]				0.774		29(23.0)			13(10.3)		42(33.3)[25.0-41.7]				0.073		
1–15		49(24.5)	17(8.5)	66(33.0)[26.4–39.6]				41(20.5)			12(6.0)		53(26.5)[20.3–32.7]						60(30.0)			32(16.0)		92(46.0)[39.0–53.0]						
> 15		63(26.5)	22(9.2)	85(35.7)[29.6–41.8]				54(22.7)			7(2.9)		61(25.6)[20.0-31.2]						71(29.8)			24(10.1)		95(39.9)[33.6–46.2]						
Number of outpatients treated in half a day																														
0		65(29.1)	20(9.0)	85(38.1)[31.7–44.5]^a^			0.046	47(21.1)			12(5.4)		59(26.5)[20.6–32.3]				0.051		77(34.5)			35(15.7)		112(50.2)[43.6–56.8]^a^				< 0.001		
1–15		25(26.3)	9(9.5)	34(35.8)[26.0-45.6]^ab^				28(29.5)			4(4.2)		32(33.7)[24.0-43.4]						27(28.4)			10(10.5)		37(38.9)[29.0-48.9]^ab^						
> 15		47(19.1)	21(8.5)	68(27.6)[22.0-33.3]^b^				41(16.7)			11(4.5)		52(21.1)[16.0-26.3]						56(22.8)			24(9.8)		80(32.5)[26.6–38.4]^b^						
Providing psychological counseling or not?																														
No		94(23.0)	35(8.6)	129(31.5)[27.0-36.1]			0.186	84(20.5)			16(3.9)		100(24.4)[20.3–28.6]				0.422		117(28.6)			49(12.0)		166(40.6)[35.8–45.4]				0.990		
Yes		43(27.7)	15(9.7)	58(37.4)[29.7–45.1]				32(20.6)			11(7.1)		43(27.7)[20.6–34.9]						43(27.7)			20(12.9)		63(40.6)[32.8–48.5]						
Forms of attacks that have been suffered																														
Never been attacked		14(27.5)	1(2.0)	15(29.4)[16.5–42.4]^a^			0.034	9(17.6)			1(2.0)		10(19.6)[8.3–30.9]^ab^				0.035		21(41.2)			4(7.8)		25(49.0)[34.8–63.2]				0.184		
Suffered only one form of attack		54(21.2)	17(6.7)	71(27.8)[22.3–33.4]^a^				48(18.8)			8(3.1)		56(22.0)[16.8–27.1]^b^						70(27.5)			27(10.6)		97(38.0)[32.0–44.0]						
Suffered two forms of attack		55(25.7)	26(12.1)	81(37.9)[31.3–44.4]^a^				43(20.1)			16(7.5)		59(27.6)[21.5–33.6]^ab^						57(26.6)			27(12.6)		84(39.3)[32.7–45.8]						
Suffered three or more forms of attack		14(31.8)	6(13.6)	20(45.5)[30.1–60.8]^a^				16(36.4)			2(4.5)		18(40.9)[25.8–56.0]^a^						12(27.3)			11(25.0)		23(52.3)[36.9–67.6]						
Perceived stress																														
Low		82(20.3)	8(2.0)	90(22.3)[18.2–26.4]			< 0.001	51(12.7)			0(0.0)		51(12.7)[9.4–15.9]				< 0.001		93(23.1)			10(2.5)		103(25.6)[21.3–29.8]				< 0.001		
High		55(34.2)	42(26.1)	97(60.2)[52.6–67.9]				65(40.4)			27(16.8)		92(57.1)[49.4–64.9]						67(41.6)			59(36.6)		126(78.3)[71.8–84.7]						
Social support																														
Low		31(38.8)	17(21.3)	48(60.0)[49.0–71.0]^a^			< 0.001	25(31.3)			12(15.0)		37(46.3)[35.1–57.4]^a^				< 0.001		31(38.8)			23(28.7)		54(67.5)[57.0–78.0]^a^				< 0.001		
Moderate		90(23.4)	33(8.6)	123(32.0)[27.3–36.7]^b^				77(20.1)			15(3.9)		92(24.0)[19.7–28.2]^b^						110(28.6)			44(11.5)		154(40.1)[35.2–45.0]^b^						
High		16(16.0)	0(0.0)	16(16.0)[8.7–23.3]^c^				14(14.0)			0(0.00)		14(14.0)[7.1–20.9]^b^						19(19.0)			2(2.0)		21(21.0)[12.9–29.1]^c^						

*χ*^*2*^ tests were used to compare the prevalence of mild-to-severe symptoms of depression, anxiety, and burnout in different populations. In variables with three or more classifications, there was no statistically significant difference between the two classifications with the same letters in the upper corner (*P* ≥ adjusted α Level), and the difference was statistically significant between the two classifications without the same letters in the upper corner (*P* < adjusted α Level). According to the Bonferroni method, the adjusted α Level was 0.05/ (number of comparisons)

### Risk factors associated with depression, anxiety, and burnout

All the statistically significant variables in the Chi-square test were entered into the multivariate analysis (results in Table 3). It is particularly important that the psychiatrists who perceived higher stress were more likely to suffer from depressive symptoms (adjusted ORs: 4.431 [95%CI, 2.907–6.752]); anxiety symptoms (adjusted ORs: 8.280 [95%CI, 5.255–13.049]), and burnout (adjusted ORs: 9.102 [95%CI, 5.795–14.298]). In contrast, receiving high social support was an independent protective factor against symptoms of depression (adjusted ORs: 0.176 [95%CI, [0.080–0.386]), anxiety (adjusted ORs: 0.265 [95%CI, 0.111–0.630]) and burnout (adjusted ORs 0.319 [95%CI, 0.148–0.686]). In the multivariable analysis, having a medium annual income (14,360–43,080 $ ) was found to be associated with a lower risk for the symptoms of depression (adjusted ORs: 0.467 [95%CI, 0.292–0.784]) and anxiety (adjusted ORs: 0.524 [95%CI, 0.314–0.874]). Working in the field of psychiatry for more than 10 years (adjusted ORs: 1.948 [95%CI, 1.244–3.049]), and working in a secondary hospital (adjusted ORs: 2.427[95%CI, 1.045–5.637]) was related to the higher risk for depression symptoms. Having children displayed a remarkably higher risk for anxiety (adjusted ORs: 2.973 [95%CI, 1.732–5.104]). Intermediate or above professional title was a protective factor for burnout (adjusted ORs for Intermediate title: 0.479 [95%CI, 0.302–0.758]; adjusted ORs for senior title: 0.561 [95%CI, 0.337–0.933]).

**Table 3:** Multivariable Regression Analysis of Risk Factors Associated with Symptoms of Depression, Anxiety, and Burnout among Psychiatrists in Beijing, China

Variables		Depression^a^				Anxiety^b^				Burnout^c^		
	B	SE	AOR(95%CI)	*P* value	B	SE	AOR(95%CI)	*P* value	B	SE	OR(95%CI)	*P* value
Having children or not												
No	NA	NA	NA	NA			1[Reference]		NA	NA	NA	NA
Yes	NA	NA	NA	NA	1.090	0.276	2.973(1.732–5.104)	< 0.001	NA	NA	NA	NA
Annual income,												
≤ 14,360			1[Reference]				1[Reference]		NA	NA	NA	NA
> 14,360 ≤ 43,080	-0.761	0.240	0.467(0.292–0.748)	0.002	-0.647	0.261	0.524(0.314–0.874)	0.013	NA	NA	NA	NA
> 43,080	-0.123	0.391	0.884(0.411–1.903)	0.753	-0.252	0.395	0.777(0.359–1.684)	0.523	NA	NA	NA	NA
Professional title												
Primary title	NA	NA	NA	NA	NA	NA	NA	NA			1[Reference]	
Intermediate title	NA	NA	NA	NA	NA	NA	NA	NA	-0.737	0.234	0.479(0.302–0.758)	0.002
Senior title	NA	NA	NA	NA	NA	NA	NA	NA	-0.578	0.260	0.561(0.337–0.933)	0.026
Level of medical institution												
Primary medical institutions			1[Reference]		NA	NA	NA	NA	NA	NA	NA	NA
Secondary hospital	0.887	0.430	2.427(1.045–5.637)	0.039	NA	NA	NA	NA	NA	NA	NA	NA
Tertiary hospital	0.306	0.421	1.358(0.595–3.097)	0.468	NA	NA	NA	NA	NA	NA	NA	NA
Working years in psychiatry, y												
≤ 10			1[Reference]		NA	NA	NA	NA	NA	NA	NA	NA
> 10	0.667	0.229	1.948(1.244–3.049)	0.004	NA	NA	NA	NA	NA	NA	NA	NA
Perceived stress												
Low			1[Reference]				1[Reference]				1[Reference]	
High	1.489	0.215	4.431(2.907–6.752)	< 0.001	2.114	0.232	8.280(5.255–13.049)	< 0.001	2.209	0.230	9.102(5.795–14.298)	< 0.001
Social support												
Low			1[Reference]				1[Reference]				1[Reference]	
Moderate	-0.908	0.288	0.403(0.229–0.709)	0.002	-0.799	0.315	0.450(0.243–0.834)	0.011	-0.531	0.304	0.588(0.324–1.066)	0.080
High	-1.739	0.402	0.176(0.080–0.386)	< 0.001	-1.328	0.442	0.265(0.111–0.630)	0.003	-1.143	0.391	0.319(0.148–0.686)	0.003

B means regression coefficient; SE, standard error; AOR, adjusted odds ratio; CI, confidence interval;NA, not available (Variables that were not included in the regression equation due to no statistically significant differences during the chi square test or when selecting independent variables through the forward LR method);

^a^ Depression was defined as Patient Health Questionnaire-9 score of 5 or higher

^b^ Anxiety was defined as Generalized Anxiety Disorder-7 score of 5 or higher

^c^ Burnout was defined as one or more dimensions in Maslach Burnout Inventory-General Survey score of 3 or higher

## Discussion

To the best of our knowledge, this is the first study that systematically investigated the prevalence of and risk factors associated with mental health symptoms (i.e., symptoms of depression, anxiety, and burnout) by standardized rating scales among psychiatrists during the COVID-19 pandemic in China. We found that approximately one-third of the psychiatrists exhibited depression, one-quarter showed anxiety symptoms, and more than 40% experienced burnout. We also identified several risk factors. Having a medium annual income was associated with a lower risk for the symptoms of depression and anxiety. Levels of the medical institution and working duration in psychiatry were related to depression symptoms. Having children was a risk factor for anxiety symptoms. Psychiatrists with lower professional titles were more likely to suffer from burnout. Specifically, perceived stress and social support were associated with these three mental health issues. These findings provide a comprehensive profile of the psychological status of the psychiatrist in Beijing, China, during the period when the COVID-19 epidemic continues to affect and call for action in delivering appropriate training to protect mental health and developing appropriate interventions for psychiatrists.

The present survey shows that during the prevalence of COVID-19, a considerable proportion of psychiatrists is experiencing mental health conditions, which is similar to previous studies on healthcare worker’s [41, 42]. In contrast to previous studies, we detected a higher prevalence of depressive symptoms than anxiety. A recent survey also found similar results that the prevalence of depressive symptoms is higher than anxiety in Mexican psychiatrists [43]. One of the important reasons may be that in the early stage of the COVID-19 epidemic, physicians often showed anxiety-related behaviors, such as tension, worry, and uneasiness. With the persistence of the epidemic, the awareness of the epidemic gradually improved. However, the work pressure related to the epidemic persists, such as being unable to visit relatives for a long time. The initial worry gradually decreased and was internalized into a continuous depressive reaction, including depression emotion, pessimism, and despair [44]. Another reason may be due to the self-diagnosis process, as a previous study suggested that physicians tend to under- or over-react to their symptoms, switching from diagnosing diseases with the worst prognosis to ruling out a disorder altogether [45]. A similar problem may happen to the psychiatrist as well. However, data on anxiety and depression in psychiatrists are very insufficient, making it impossible to conclude the impact of the pandemic on the mental health conditions in psychiatrists [28]. The burnout rate in our study is consistent with a recently published report, which showed that 42% of psychiatrists reported burnout in the last year [46]. Plenty of studies have investigated burnout in doctors, including the psychiatrist [47]. It is often reported that psychiatrists have a lower burnout rate than other specialists [48, 49]. A previous study also reported a lower burnout rate (38.4%) among psychiatrists than other specialists in China [12]. According to the newly released Medscape Psychiatrist Lifestyle, Happiness & Burnout Report 2022, about half of psychiatrists (52%) reported that they were more burned out now than during the initial quarantine months of the pandemic [46].

In the present study, we found that having a medium annual income was associated with a lower risk for the symptoms of depression and anxiety. This is to some extent consistent with Western research, which suggest that lower-income Americans are less likely to report being in good health and are more likely to have high-stress levels [50]. Depression and anxiety may affect people’s cognitive function, affecting workability and thus economic income [51]. Conversely, poverty itself can influence cognitive function by capturing attention and taxing mental bandwidth [52] In addition, depression and anxiety may affect an individual’s economic level by influencing their beliefs, preferences, creativity, work hours, efficiency, creativity, etc. [51]. We also found that working in a Secondary hospital, and working longer in the psychiatry field increases the risk of depressive symptoms. This may be related to the shortage of medical staff in secondary hospitals, higher work pressure, and relatively low income. On the other hand, the longer the working experience, the more work tasks one needs to undertake, such as undertaking routine medical work, administrative management work, organizing and attending various academic conferences, and so on. These often occupy many psychiatrists’ spare time, consume a lot of energy, and increase occupational stress, which may affect depression [46]. In the present survey, having children increase the risk of anxiety symptoms. It is consistent with a recently published study which suggested that having children increased daily stress in the general population [53], and parents experience greater anxiety, depression, and marriage dissatisfaction than spouses without children [54]. Many studies have shown that parents’ anxiety levels increase from preparation for pregnancy to birth and during the care of the baby [54, 55]. Additionally, we found that psychiatrists with lower professional titles were more likely to suffer from burnout. A recently published meta-analysis also reported a negative effect of lower professional levels on mental health in healthcare workers [56]. Lower professional titles often mean less experience in treating patients and facing complex situations, undertake more detailed, specific, and trivial tasks, which may increase individual exhaustion.

High perceived stress, which scored more than 25 in CPSS, was an independent risk factor for depression, anxiety, and burnout. Consistent with previous studies, perceived stress also increased the risk of depressive symptoms in interns during medical internships [57]. An extensive survey of psychiatrists identified “out-of-hours of duty, dealing with difficult and hostile relatives of patients, arranging admissions, paperwork, balancing personal and professional lives, and managing suicidal or homicidal patients” as particularly stressful experiences [46]. After the outbreak of COVID-19, the Chinese government adopted strict prevention and control measures different from those of most countries in the world so that the development of the epidemic in China did not get out of control. These measures put forward higher requirements for medical staff, encouraging them to adopt the two-dot and one-line lifestyle (only going to and from the hospital and residence, not going to other places, especially not participating in gathering social activities), irregular support for epidemic prevention and control in different regions, and support for large-scale nucleic acid testing of a novel coronavirus, which may make Chinese doctors face higher work pressure, and make their mental health status different from other countries during this period. It is worth noting that about 91% of the psychiatrist have been assaulted at least once in the present study, which suggests that psychiatrists have more work-related emotional exhaustion [58], and their work is more emotionally difficult than that of other doctors. A previous study showed that insomnia mediated the association between perceived stress and depression [59]. Another study showed that alexithymia moderated the perceived stress-mediated relations between the pandemic events and the changes in depressive and anxiety symptoms by enhancing the detrimental effect of perceived stress on mental health [60]. Effective and acceptable programs should be implemented to reduce the perceived stress in the psychiatrist. Tai Chi/Yoga could be an alternative method for stress reduction for individuals who live under high stress or negative emotions [61].

High social support scores of more than 49 in SSRS were an independent protective factor for depression, anxiety, and burnout. The existing empirical literature proposes that social support directly influences the well-being of medical staff [62, 63]. A recently published study reported a moderating effect of social support on the relationship between burnout and anxiety symptoms among intensive care unit staff [64]. It has been well-documented that social support can protect people from mental health problems [65]. A recent meta-analysis revealed that in Asia, good social support is related to decreased depression [66]. Social support, particularly family support, is an important social factor in geriatric depression in China [67]. Seeking social support as a means of coping with adversity has been categorized as a problem-focused coping strategy and has been found to effectively reduce stress [68]. During the pandemic, when stress and anxiety are elevated, adequate social support may help healthcare workers maintain healthy emotional states [69]. Considering the significant protective effect of social support, formal support systems are needed to provide extra support for psychiatrists during life events to decrease the risk of mental problems and burnout.

To the best of our knowledge, this is the first study that systematically investigated the prevalence and risk factors associated with mental health symptoms by standardized rating scales among psychiatrists during the COVID-19 pandemic in China. The present study has several limitations. First, measures were obtained from self-reports rather than clinical diagnoses and may reflect bias in reporting. Second, this was a cross-sectional study. Therefore, associations between mental health symptoms and risk factors cannot necessarily be considered causal relationships. Third, The results may only reflect the mental health status of psychiatrists in Beijing when the COVID-19 epidemic continued for two years. However, due to the lack of data in the early stage of the pandemic, we cannot obtain the exact impact of different stages of the COVID-19 epidemic on the mental health of psychiatrists. Follow-up nationwide studies are needed to determine the possible long-term mental health outcomes in psychiatrists associated with the COVID-19 pandemic in China.

## Conclusions

The prevalence of symptoms of depression, anxiety, and burnout was worth noting in this sample of psychiatrists from Beijing, China, during the COVID-19 pandemic. Perceiving more stress and receiving lower social support were independent risk factors for these three mental health issues. Besides recruiting more future psychiatrists, the retention issues of currently practicing psychiatrists are more urgent in the shortage of psychiatrists. Our findings may provide helpful information for policymakers to help them make strategies to protect mental health and develop appropriate interventions for psychiatrists, and we proposed that effective and acceptable programs to reduce stress and increase social support are important for psychiatrists’ mental health and well-being.

## Electronic supplementary material

Below is the link to the electronic supplementary material.

Supplementary Material 1

## Data Availability

The data presented in this study are available on request from the corresponding author. The data are not publicly available due to ethical reasons.
