# Supplementary material for: Depression, anxiety, and burnout among psychiatrists during the COVID-19 pandemic: a cross-sectional study in Beijing, China
Source: BMC Psychiatry. 2023 Jul 10;23:494. doi: 10.1186/s12888-023-04969-5 (PMC10334525; doi:10.1186/s12888-023-04969-5)
Supplement: Supplementary file 1 — Supplementary Material 1 [file 12888_2023_4969_MOESM1_ESM.docx]

**Supplemental Materials**

**Depression, Anxiety, and Burnout among Psychiatrists during the COVID-19 Pandemic: A Cross-sectional Study in Beijing, China**

Ping Dong^1, #^; Xiao Lin^1, #^; Fei Wu^1^; Sijia Lou^1^; Na Li^1^; Sifan Hu^1^; Le Shi^1^; Jia He^1^; Yundong Ma^1^; Yanping Bao^2^; Lin Lu^1, 2, 3^; Wei Sun^1, *^; Hongqiang Sun^1, *^

^1^ Peking University Sixth Hospital, Peking University Institute of Mental Health, NHC Key Laboratory of Mental Health (Peking University), National Clinical Research Center for Mental Disorders (Peking University Sixth Hospital), Peking University, Beijing 100191, China

^2^ National Institute on Drug Dependence and Beijing Key Laboratory on Drug Dependence Research, Peking University, Beijing 100191, China

^3^ Peking-Tsinghua Center for Life Sciences and PKU-IDG, McGovern Institute for Brain Research, Beijing 100191, China

# Equal Author Contribution:

Ping Dong and Xiao Lin contributed equally as the first authors.

*Corresponding authors:

Hongqiang Sun, MD, PhD, Peking University Sixth Hospital, Peking University Institute of Mental Health, 51 Huayuanbei Road, Beijing 100191, China.

Tel: +86-10-8280 5960; Fax: +86-10-6202 6310; E-mail: sunhq@bjmu.edu.cn

OR

Wei Sun, MD, Peking University Sixth Hospital, Peking University Institute of Mental Health, 51 Huayuanbei Road, Beijing 100191, China.

Tel: +86-10-6272 3844; E-mail: weisun@bjmu.edu.cn

**The regression model**

The variables in Table 2, with statistical significance after the chi-square test, were included as possible influencing factors in the logistics regression analysis, and the independent variables were screened by the forward LR method to construct a regression model. In the logistics regression analysis of depression, the independent variables that finally entered the regression equation were: Annual income, Level of the medical institution, Working years in psychiatry, Perceived stress, and Social support, and the p-value of the likelihood ratio test <0.001, and we also assessed the goodness of fit of the logistic regression model (P=0.633). In the logistics regression analysis of anxiety, the independent variables entering the regression equation were Children, Annual income, Perceptual stress, and Social support, and the p-value of the likelihood ratio test <0.001, and we also assessed the goodness of fit of the logistic regression model (P=0.999). In the logistics regression analysis of burnout, the independent variables that finally entered the regression equation were Professional title, Perceptual pressure, and Social support, and the p-value of the likelihood ratio test <0.001, and we also assessed the goodness of fit of the logistic regression model (P=0.954).

eTable 1. Summary of the Questionnaire Related to This Study

| **Questions** | **Types** | **Choices** |
| --- | --- | --- |
| **Part One: Demographic information** | | |
| Do you agree to participate in this study? | Single choice | Yes/No |
| Are you a psychiatrist working in Beijing? | Single choice | Yes/No |
| Do you have a history of mental illness met the criteria on DSM-5 (Schizophrenia/Bipolar disorder/Depressive disorder/Anxiety disorder/Obsessive-compulsive disorder/insomnia disorder/alcohol dependence/drug dependence)? | Single choice | Yes/No |
| Please select your gender: | Single choice | Male/Female |
| Please fill in your age(years): | Blank | / |
| Please select your marital status: | Single choice | Married/Unmarried/ Divorced/ Widowed |
| How many children do you have? | Single choice | 0/1/2/3/≥4 |
| Please select your highest educational level: | Single choice | Junior college education or below/ Bachelor degree/ Master degree/ Doctoral degree |
| Please select your annual income (ten thousand yuan): | Single choice | ≤5/>5≤10/>10≤20/>20≤30/>30≤40/>40 |
| **Part Two**: **Questions about the professional lives** | | |
| Please select your professional title: | Single choice | Primary title (Resident or below)/ Intermediate title (Attending doctor)/ Senior title (Associate chief physician/ Chief physician) |
| Please select the category of medical institution where you work: | Single choice | Psychiatric hospital/ General hospital/ Other specialized hospitals/ Other medical institutions |
| Please select the level of medical institution where you work: | Single choice | Primary medical institutions / Secondary hospital /Tertiary hospital |
| Please choose your major in psychiatry: | Single choice | General psychiatry/ Geriatric psychiatry/ Pediatric psychiatry/ Addictive psychiatry/ Sleep medicine/ Mental rehabilitation/ Psychosomatic medicine/ Emergency psychiatry/Others |
| Please fill in your current work area: | Blank | / |
| How many years have you worked in psychiatry? | Blank | / |
| How many days do you work on average per week? | Blank | / |
| How many hours do you work on average every day? | Blank | / |
| How many beds are you responsible for on average in the ward? | Blank | / |
| How many outpatients do you treat on average in half a day? | Blank | / |
| Do you provide psychological counseling (refer to those who last for 30 minutes or more each time, and use professional psychological counseling or psychotherapy technology for evaluation and intervention)? | Single choice | Yes/No |
| What forms of attacks have you suffered from patients and their families during your practice of psychiatry? | Multiple choice | Never been attacked/ Verbal threats / Attack with limbs/ Attack with instruments/ Others |

eTable 2. Factors Associated with Symptoms of Depression, Anxiety, and Burnout Included in the Regression Model.

| **Factors** | **Definitions** | **Types of variables** | **Reference category** | **Summarizing forms** |
| --- | --- | --- | --- | --- |
| Gender | In the following 2 categories: 1. male; 2. female | Categorical | male | Odds ratio with a 95% confidence interval |
| Age | In the following 3 categories: 1. 18-35; 2. 36-45; 3.46-65 | Categorical | 18-35 | Odds ratio with a 95% confidence interval |
| Marital status | In the following 2 categories: 1. married; 2. unmarried or divorced | Categorical | married | Odds ratio with a 95% confidence interval |
| Having children or not | In the following 2 categories: 1. No; 2. Yes | Categorical | No | Odds ratio with a 95% confidence interval |
| Educational level | In the following 3 categories: 1. bachelor degree or below; 2. master degree; 3. doctoral degree | Categorical | bachelor degree or below | Odds ratio with a 95% confidence interval |
| Annual income($) | In the following 3 categories: 1. ≤14360; 2. >14360≤43080; 3. >43080 | Categorical | ≤14360 | Odds ratio with a 95% confidence interval |
| Professional title | In the following 3 categories: 1. primary title; 2. intermediate title; 3. senior title | Categorical | primary title | Odds ratio with a 95% confidence interval |
| Category of medical institution | In the following 2 categories: 1. psychiatric hospital; 2. other medical institutions | Categorical | psychiatric hospital | Odds ratio with a 95% confidence interval |
| Level of medical institution | In the following 3 categories: 1. primary medical institutions; 2. secondary hospital; 3. tertiary hospital | Categorical | primary medical institutions | Odds ratio with a 95% confidence interval |
| Working years in psychiatry | In the following 2 categories: 1. ≤10; 2. >10 | Categorical | ≤10 | Odds ratio with a 95% confidence interval |
| Working days per week | In the following 2 categories: 1. ≤5; 2. >5 | Categorical | ≤5 | Odds ratio with a 95% confidence interval |
| Daily working hours | In the following 2 categories: 1. ≤8; 2. >8 | Categorical | ≤8 | Odds ratio with a 95% confidence interval |
| Number of beds in charge | In the following 3 categories: 1. 0; 2. 1-15; 3. >15 | Categorical | 0 | Odds ratio with a 95% confidence interval |
| Number of outpatients treated in half a day | In the following 3 categories: 1. 0; 2. 1-15; 3. >15 | Categorical | 0 | Odds ratio with a 95% confidence interval |
| Providing psychological counseling or not | In the following 2 categories: 1. No; 2. Yes | Categorical | No | Odds ratio with a 95% confidence interval |
| Forms of attacks that have been suffered | In the following 4 categories: 1. never been attacked; 2. suffered only one form of attack; 3. suffered two forms of attack; 4. suffered three or more forms of attack | Categorical | never been attacked | Odds ratio with a 95% confidence interval |
| Perceived stress | In the following 2 categories: 1. low; 2. high | Categorical | low | Odds ratio with a 95% confidence interval |
| Social support | In the following 3 categories: 1. low; 2. moderate; 3. high | Categorical | low | Odds ratio with a 95% confidence interval |
